# The Association Between new Nurses’ Gerontological Education, Personal Attitudes Toward Older Adults, and Intentions to Work in Gerontological Care Settings in Ontario, Canada

**DOI:** 10.1177/08445621211063702

**Published:** 2021-12-29

**Authors:** Jessica Smith, Monakshi Sawhney, Lenora Duhn, Kevin Woo

**Affiliations:** 1 School of Nursing, 12363 Faculty of Health Sciences, Queen’s University, Kingston, Canada; 2 School of Nursing & Department of Anesthesiology and Perioperative Medicine, Faculty of Health Sciences, 4257 Queen’s University, Kingston, Canada

**Keywords:** Registered nurse, practical nurse, career choice, gerontological care setting, long term care, geriatric unit

## Abstract

**Background:**

The older adult population in Canada is increasing, and many will require care within an acute geriatric unit (AGU) or long-term care facility (LTCF). However, the nursing workforce is not growing at the same pace as the population is aging. New graduate nurses may be able to fill this gap; therefore, it is important to understand their intentions of working in gerontological care settings (i.e., AGU or LTCF).

**Aim:**

To examine if nursing education and personal attitudes toward older adults influence newly registered nurses’(RNs) intentions to work in a gerontological care setting.

**Method:**

Nurses (n= 1,103) who registered with the College of Nurses of Ontario for the first time in 2018 were invited to complete a questionnaire.

**Results:**

The majority of participants (n = 181) reported a positive attitude toward older adults. However, only 14% reported an intention to work in a gerontological care setting. Participants who completed multiple geriatric focused clinical placements were more likely to report an intention to work in these settings.

**Conclusion:**

This study provides some information regarding the attitudes and intentions of newly RNs toward a career in gerontological care settings. Further research is needed to understand nurses’ intentions regarding working in AGUs or LTCFs.

## Background and purpose

The older adult population in Canada is projected to increase by 68% within the next 20 years (Canadian Institute for Health Information [CIHI], 2017). This population of older adults will require nursing care within a long-term care (LTC) facility or specialized setting, such as an acute geriatric unit (AGU). There are approximately 32,000 Ontario residents waiting for a LTC placement (Ontario Long Term Care Association, 2018). While the population of older adults in Canada (age 65 + ) is steadily increasing, the nursing workforce required to care for this aging population is only growing at a rate of 1.7%; the lowest growth rate in the past 10 years (CIHI, 2019). In Ontario, Canada 16.7% of the population is over the age of 65 years, with a projected increase to 24.8% by the year 2041 (Ontario Ministry of Finance, 2018). The nursing workforce needs to continue to grow to ensure there is an adequate workforce available to care for older adults. For this reason, it is important to understand if registered nurses (RNs) and registered practical nurses (RPNs) in Ontario have an intention to work in gerontological care settings.

Gerontological nursing involves providing care and support for the older person and their family, including providing support for aging well, promotion of geriatric mental health, illness prevention, management of chronic disease, and the implementation of dementia-friendly initiatives (Canadian Gerontological Nurses Association, 2010). Although older adults receive care in many settings, in this paper, the term gerontological care setting will be used to describe AGU, and long-term care facility (LTCF). In these settings nurses who specialize in gerontology can advocate for the needs of older adults, encourage their well-being and enhance their functional abilities (Canadian Gerontological Nurses Association, 2010). Both RNs and RPNs are members of the interprofessional teams that work in AGUs and LTCFs in Ontario. RPN is the class of nursing registration used in Ontario for Practical Nurses. Their role is similar to licensed practical nurse (LPN), which is the title used in other Canadian Provinces and the United States (Canadian Nurses Association, 2019; National Council of State Boards of Nursing, 2019). Unfortunately, gerontological nursing has been ranked as either the second to least preferred or least preferred nursing specialty for postgraduation employment (Birks et al., 2014; Happell, 2002; King et al., 2013; Stevens, 2011; Stevens & Crouch, 1995). Emergency department nursing, pediatric/neonatal nursing, and intensive care nursing are the most preferred specialty areas for employment (Birks et al., 2014; Happell, 2002; King et al., 2013; Stevens, 2011; Stevens & Crouch, 1995).

There are major differences in delivery of gerontological nursing content at Canadian Universities, including when in the curriculum students receive their gerontological theory education, and how this curriculum is taught regarding stand-alone courses, integrated material, and clinical placement settings (Dahlke et al., 2019; McCleary et al., 2017; Madueke et al., 2020; Prentice, 2012). In Ontario, 50% of nursing programs include a gerontological theory course that students are required to complete prior to graduation (McCleary et al., 2017). One of the limiting factors in the delivery of gerontological theory or clinical placements is having faculty or instructors with expertise in gerontological nursing. Even when Schools of Nursing do have faculty with expertise in gerontological nursing, only 50% report that their graduates have the necessary competencies to provide seniors’ care (McCleary et al., 2017).

The quality and timing of geriatric focused clinical rotations and gerontological theory courses students receive can influence their attitudes toward older adults and toward working in a gerontological care setting following graduation (Dahlke et al., 2019; Haron et al., 2013; Hsu et al., 2018; Madueke et al., 2020; Prentice, 2012; Shen & Xiao, 2012). Nursing students who complete a stand-alone gerontological nursing theory course are more likely to consider a career in this area (Haron et al., 2013; King et al., 2013), and if students report a high satisfaction with their gerontological theory course, or gerontological clinical placement their attitude toward older adults is significantly better (Hsu et al., 2018; Shen & Xiao, 2012). Gerontological clinical placements completed in the first year of nursing school can be positively influenced by clinical instructors who have gerontological nursing experience, by clinical instructors and nurses who have positive attitudes toward older adults, and if the gerontological care settings is a welcoming and positive environment (Dahlke et al., 2019; Haron et al., 2013; Sheffler, 1995, 1998). Completing gerontological clinical placements in the third or fourth years can positively influence students toward a future career working in either LTCFs or AGUs. A longitudinal, mixed-methods study conducted in the United States examined the role gerontological nursing education has on 80 students’ attitudes toward older adults and their future work preferences (King et al., 2013). These students completed two geriatric-focused clinical placements in during their third year of school, and completed a stand-alone gerontological theory course in their fourth year. The students’ attitudes toward older adults improved throughout their program, and gerontological care settings increased in preference as a specialty area for postgraduation employment (King et al., 2013).

Past employment experiences and feeling unprepared to care for older adults has a negative impact on nursing students. Past experience working in LTCFs as a personal support worker (PSW) or completing the first clinical placement in a LTCF can negatively influence nursing students’ attitudes toward older adults and future postgraduation employment in these settings if they do not have positive role models (Dahlke et al., 2019; Happell, 2002; Prentice, 2012; Stevens, 2011). This is due to the fact that nursing students see their role in LTCFs or AGUs as being the same as the PSW role, and they lack the ability to manage the complexity of providing care for people with multiple comorbid conditions or dementia behaviours (Dahlke et al., 2019; Madueke et al., 2020). Nursing students may also perceive working in a gerontological care setting as an infringement on future career advancements (Happell, 2002; Stevens, 2011; Stevens & Crouch, 1995). Despite theoretical education and clinical placements, students report that they prefer not to work in gerontological care settings. This lack of interest in working in LTCFs or AGUs is due to their low confidence in this area of nursing, the nature of the work, the work environment, and their understanding of a negative wage gap between nurses working in gerontological care settings compared to nurses working in acute care settings (Dahlke et al., 2019; Hsu et al., 2018; King et al., 2013; Madueke, et al., 2020; Shen & Xiao, 2012).

The factors most predictive of students choosing a career in gerontological nursing include having a positive attitude toward older adults, having the opportunity to expand their nursing role to a clinical nurse specialist, having positive role models, and positive pre-training experiences (theoretical and clinical) with older adults (Haron et al., 2013). However, little is known about the attitudes toward older adults, gerontological nursing education, current employment in either a LTCF or AGU, and employment preferences of newly RNs in Ontario, Canada. Therefore, the aim of this study was to examine how nursing education and personal attitudes toward older adults influence newly RNs’ intentions to work in a gerontological care setting such as LTCF or AGU. The primary research question for this study was: Is there a relationship between gerontological nursing education and personal attitudes toward older adults, in relation to newly registered RNs’ and RPNs’ intentions to work in gerontological care settings in Ontario, Canada? Additional questions included: Does the existing nursing curriculum equip newly registered RNs and RPNs in Ontario, Canada, with the knowledge, skill, and judgement to care for the older adult population?; What are newly registered RNs’ and RPNs’ personal attitudes toward older adults?; Do newly registered RNs and RPNs in Ontario, Canada, intend to work in a gerontological care setting within the next 5 years?

## Method

### Research design

This cross-sectional study included RNs and RPNs (n = 1,103) who registered with the CNO for the first time in 2018, and were able to read and write English. RNs and RPNs were excluded if they had previously worked as a nurse or had been registered with any other provincial regulatory body in Canada prior to 2018. Ethics approval was obtained from the relevant ethics board (NURS-463-18; TRAQ #6025599) and the CNO.

### Recruitment and data collection

The sample size estimate for this study was based on nurses intention to work in a gerontological care setting. Haron et al. (2013) reported that 39% of nursing students would consider a career working in a gerontological care setting if they completed a clinical rotation in a LTCF or AGU. For this study, it was expected that similar results would be obtained. A sample size estimate was calculated using the program, G*Power© (Faul et al., 2009). Allowing for an alpha of 0.05, and power set at 80%, the minimum required sample was 179 participants to answer the primary research question.

To obtain the sample for this study, a list of 4654 names and mailing addresses of newly registered RNs and RPNs who consented to being contacted for research purposes was obtained from the CNO. There was no indication on the mailing list which nurses were registered as an RN or RPN. The entire list of 4654 names were entered into a Microsoft Excel© spreadsheet. Using the RAND formula in Microsoft Excel© the names were randomized. On April 3, 2019, 1,000 randomly chosen nurses were mailed an invitation to participate in the study. This invitation included the informed consent form, the first author's contact information, the questionnaire, a prepaid return stamped envelope, and an expression of gratitude for their time and consideration. Participants were not recontacted after the initial survey was mailed out.

The participants could return the completed questionnaire by mail, or complete the questionnaire online through Qualtrics© by scanning a QR code or accessing the provided website link. To increase response rates and to obtain an adequate sample size, 2 months later the next 103 randomized nurses were mailed an invitation to participate, along with the QR code and Qualtrics© link. Due to time and cost restraints, snowball recruitment was also utilized. New graduate RPNs and RNs in Ontario, who were known to the authors were asked to participate in this study. These nurses were emailed the survey link.

### Outcome measures

Demographic information including age, gender, ethnicity, and frequency of interaction with older adults was collected, all of which have been shown to impact attitudes toward older adults (Gorelik et al., 2000; Luo et al., 2013; Shen & Xiao, 2012). To assess for the completion of gerontological nursing education, participants were asked if they had a gerontological theory course, and/or clinical placement(s) during nursing school, and in which year(s) of study this occurred. Participants were also asked if they thought their education provided them with the knowledge, skill and judgement to care for the older adult population by answering yes, somewhat, or no.

The participant's attitudes toward older adults was assessed using the Kogan's Attitudes Toward Old People (KAOP) Scale. The KAOP Scale is a 7-point Likert scale comprising 17 positive and negative paired items about attitudes toward older adults. Scores range from 34 to 252, with higher scores indicating positive attitudes toward older adults (Matarese et al., 2013). The KAOP scale has established reliability and good internal consistency (α = 0.76–0.81) (Mansfield-Green et al., 2015; Matarese et al., 2013).

To assess the intentions of newly registered RNs and RPNs to work in gerontological care settings, a modified version of the questionnaire by Stevens and Crouch (1995) was used. Participants were asked to rank which nursing specialty they preferred to work (1 = most preferred; 10 = least preferred). They also explained their specific ranking of “Geriatric Unit” and “LTC Facility”(Stevens & Crouch, 1995). The participants in this study were presented with a list of the 10 most common nursing specialty practice areas in Ontario, as reported by the CNO (2017, p 76). These 10 nursing specialties included: acute care, emergency room, general medicine, geriatric unit, LTCF, maternal child, mental health/psychiatric/addiction, operating room, perioperative, and primary care/public health. In addition, participants were asked if in the next 5 years they would work in a LTCF or a AGU.

### Data analysis

Data were analyzed using the Statistical Package for the Social Science (SPSS©, version 25.0, IBM). Descriptive statistical analyses (means, medians, *SD*, frequencies) were performed on the study variables and participant demographics (Pallant, 2016). Age was collapsed into two categories: “born after year 1994”, and “born before the year 1994”as 1994 was the median year for this category. Participants’ answers of “somewhat” and “no” regarding their gerontological nursing education providing them with the knowledge, skill and judgement to care for the older adult population were combined to indicate that they did not have a positive perception of their gerontological nursing education. Participants’ answers of “yes” and “maybe” regarding their intentions to work in aged care in the next 5 years were combined to indicate potential interest in gerontological nursing as a future career field.

Reverse coding was performed for all odd numbered questions of the KAOP scale as these were negative statements. Independent *t*-tests were conducted with KAOP scores. Chi-square analyses were conducted with participant demographic characteristics and gerontological nursing education responses, and intention responses.

## Results

### Demographics

From the random sample of 1,103 surveys mailed, 199 surveys were completed and returned for a response rate of 18%. The snowballing recruitment method resulted in 39 participant responses resulting in 238 returned questionnaires. One hundred and eighty-one surveys were fully completed and were included for analysis (n = 112 RNs; n = 60 RPNs). The majority of participants self-identified as female, were age 25 or younger, and obtained their nursing education in Canada (Table 1). Sixty-two percent of the participants had previous work or volunteer experience in a gerontological care setting such as LTCF (n = 112); however, the majority reported they did not currently work in gerontological care setting (either LTCF or AGU).

**Table 1.** Participant demographic characteristics.

	RN	RPN
	n (%)	n (%)
Class of nursing registration	112 (61.8)	69 (38.1)
Gender		
Female	100 (55.5)	56 (31.1)
Male	11 (6.1)	12 (6.6)
Other	1 (0.5%)	1 (0.5%)
Age		
≤25 years old	71 (41.5)	20 (11.7)
>25 years old	36 (21)	44 (25.7)
Ethnicity		
White	76 (43.9)	36 (20.8)
Asian	19 (11)	14 (8)
Other	13 (7.5)	15 (8.7)
Nursing education obtained in Canada	106 (58.6)	58 (32)
Prior work/volunteer experience in gerontological care setting	70 (62.5)	42 (23.3)
Current employment in gerontological care setting		
Yes	37 (20.4)	47 (26)
Unsure	3 (1.7)	1 (0.6)
No	72 (39.7)	21 (11.6)

Notes: RN = registered nurse; RPN = reregistered practical nurse.

### Education

Most participants (*n* = 157; 86.7%) reported having a clinical placement in a gerontological care setting, and 44.8% (*n* = 69) had this clinical placement in year one of their program. Over 30% (*n* = 52) of participants reported completing a clinical placement in a gerontological care setting in multiple years of their program, with 70% (*n* = 108) of participants completing this placement in a LTCF and approximately 60% (*n* = 93) in a hospital setting.

Only 55 (43%) participants reported they completed a full credit theory course in gerontological nursing, with 42 (34.1%) participants completing a required, stand-alone gerontological nursing course, and 38 (30.9%) participants indicating that gerontological nursing material was integrated throughout their curriculum. Thirteen (10.6%) participants received gerontological nursing education through both a “stand-alone” theory course and integrated throughout their program. When asked if their nursing education provided them with the knowledge, skill and judgement to care for the older adult population, 45% of participants (RN = 50; RPN = 31) reported “yes”, 52.5% (RN = 59; RPN = 35) reported “somewhat”, and 2.2% (RN = 1; RPN = 3) reported “no”.

### Attitude

The total mean KAOP score was 166 (*SD* = 24, range: 77–227), indicating that the participants had positive attitudes toward older adults. The Cronbach's alpha value of the KAOP questionnaire was 0.87, indicating good internal consistency. There was no significant difference in the mean attitude scores between RNs (*n* = 167, *SD* = 24) and RPNs (*n* = 164, *SD* = 25; *p* = .56). The KAOP question, “Most old people need no more love and reassurance than anyone else”, was initially missing in the online version of the survey via the QR code (*n* = 52 responses affected) before it was corrected. This question was not included within the final KAOP score for these 52 participants.

### Intention

Only 14% (*n* = 26) of participants reported that they intend to work in a gerontological care setting in the next 5 years, while the majority (*n* = 96; 53%) reported that they have no intention to do so (Table 2). Among the 84 participants who reported that they are currently working in a gerontological care setting, only 27% (*n* = 23) reported that they intend to continue to work in this setting in the next 5 years, while 54% (*n* = 45) reported may continue.

**Table 2.** Demographics and gerontological nursing education compared with new nurses intentions to work in an aged care setting in the next 5 years.

		Intention		χ^2^	Ø	OR (95% CI)	df	*p*
		Yes	No					
Full credit Gerontological nursing course	Yes	24	37	0.63	0.07	0.75 (0.37, 1.52)	1	.43
	No	31	36					
Gerontological clinical rotation	Yes	80	77	7.58	0.21	3.95 (1.40, 11.1)	1	<.01**
	No	5	19					
Clinical placement in a LTC setting	Yes	60	49	7.19	0.12	2.30 (1.25, 4.26)	1	<.01*
	No	25	47					
Clinical placement in a hospital setting	Yes	48	44	2.04	−0.11	1.53 (0.85, 2.76)	1	.15
	No	37	52					
Self-identified gender	Male	12	11	0.23	0.04	1.24 (.52, 2.98)	1	.63
	Female	73	83					
Age	≥25 years	41	39	1.21	0.08	1.4 (0.77, 2.56)	1	.27
	<25 years	39	52					
Class of nursing registration	RN	39	73	17.38	−0.31	0.27 (0.14, 0.50)	1	<.01*
	RPN	46	23					
Positive perception of gerontological nursing education	Yes	36	45	0.37	0.05	0.83 (0.46, 1.5)	1	0.55
	No	48	50					
Had previous work or volunteer experience in an aged care setting	Yes	52	60	5.15	0.169	0.49 (0.26, 0.91)	1	.02*
	No	44	25					
Currently working in an aged care setting	Yes	68	16	74.47	0.649	22.1 (10.17, 48.01)	1	<.01*
	No	15	78					

Notes: LTC = long-term care RN = registered nurse; RPN = registered practical nurse.

**Significance at *p *< .01.

A significant association was found between participants’ class of nursing registration and their intention to work in a gerontological care setting in the next 5 years, with RNs being less likely than RPNs to have positive intentions (*OR* 0.27, 95% *CI* = 0.14, 0.50; *p *< .01). A positive association was also found between participants who are currently working in a gerontological care setting and their intentions to work in a gerontological care setting in the next 5 years (*OR* 22.1, 95% *CI* = 10.17, 48.01; *p *< .01). A negative association was found between participants who had previous work or volunteer experience in a gerontological care setting and their intentions to work in this setting in the next 5 years (*OR* 0.49, 95% *CI* = 0.26, 0.91; *p *< .02). No significant association was found between the participants’ gender or age and their intention to work in a gerontological care setting in the next 5 years (*p* = 0.36; *p* = .27, respectively). There was no difference in mean KAOP scores between the participants who intend (mean = 66, *SD* = 25) or do not intend (mean = 166, *SD* = 25) to work in a gerontological care setting in the next 5 years.

A positive association was found between participants’ intention to work in a gerontological care setting in the next 5 years and completing gerontological clinical placement (*OR* 3.95, 95% *CI* = 1.40, 11.1; *p *< .01), and completing this clinical placement in a LTC setting (*OR* 2.30, 95% *CI* = 1.25, 4.26; *p *< .01). No significant association was found between the participants’ intention to work in a gerontological care setting in the next 5 years and completing a full credit gerontological theory course (*p* = .43) or reporting that they have the knowledge, skill and judgement to care for the older adult population (*p* = .55).

### Future career preferences

“Geriatric unit” and “LTCF” were ranked as the least preferred specialty settings to work in for both RNs and RPNs. Twenty-seven (15%) participants ranked “LTCF” among their top three choices, with 11 (6%) participants (RN = 5; RPN = 6) ranking LTC facility as their most preferred setting to work. “Geriatric unit”was ranked in the top three choices for 19 (10.5%), with four (2%) participants (RN = 3; RPN = 1) ranking “Geriatric unit” as their most preferred setting to work. The most preferred nursing specialty settings were acute care medicine units, perioperative, and emergency department.

## Discussion

To our knowledge, this is first study in Ontario, Canada about the attitudes and intentions of new nurses (RNs and RPNs) to work in a gerontological care setting. It included responses from both levels of “entry to practice” nurses as per requirements of the CNO (RNs and RPNs). The findings of this study indicate that newly RNs in Ontario (RNs and RPNs) have positive attitudes toward older adults. Most participants reported completing a clinical placement in a gerontological care setting, with only half of reporting that they completed a gerontological nursing theory course during their nursing education. There was no significant difference in attitude scores between the different classes of nursing registration, RN when compared to RPN (practical nurse). Despite their positive attitudes and nursing education, the majority of participants have no intention to specialize in gerontological nursing or work in a gerontological care setting. Unfortunately, the majority of nurses who were currently working in these settings reported they did not intend to continue to work in this setting.

Researchers have reported that completing a theory-based course and clinical placements focused on gerontological nursing positively influences nursing students to a career in gerontological nursing, especially if they report high satisfaction with the course or placement (Haron et al., 2013; King et al., 2013; Hsu et al., 2018). In this study, completing a clinical placement in a LTCF was positively associated with future intention to work in a gerontological care setting. However, there was no significant association was found between new graduate nurses’ report of completing a theory-based course focused on gerontological nursing and their intention to work in this field of nursing.

Interestingly, only 50% of new graduate nurses in this study reported completing a gerontological nursing theory course. This matches the results of a survey conducted in Ontario of health sciences and social sciences faculties where 46.6% of faculties reported having a required course regarding gerontology (McCleary et al., 2017). There is a need to improve the curriculum regarding the care of older adults and increase the number of faculty with expertise in gerontology (McCleary et al., 2017). There is also a need to provide students with active, positive learning opportunities with older adults to help students be better prepared to care for older adults with multiple comorbid illness and the adverse effects of these conditions such as dementia. Newly registered RNs and RPNs who were included in this study perceived their gerontological nursing education as providing them with the knowledge, skill and judgement to care for the older adult population. However, there is room for improvement given 52% of participants reported only feeling “somewhat” prepared. It is unclear if integrating gerontological theory content throughout other courses affected the participants’ feelings of preparedness to care for older adults. It is commonly found that nursing students who have more gerontological theory content or clinical experiences throughout their curriculum have better attitudes compared to those who do not (Ghimire et al., 2019; Haron et al., 2013; Hsu et al., 2018; Sheffler, 1995, 1998). However, the method of gerontological content delivery may be less important than its timing within the nursing program, and educators should strive to have geriatric-focused clinical placements align with theoretical content (Hirst et al., 2012). Ultimately, making this content explicit within the nursing curriculum will show its importance and aid in preparing students to care for older adults. Having geriatric-focused clinical placements may allow nursing students to overcome their preconceived notions about gerontological nursing and become more positive about a career in this field (King et al., 2013). Therefore, providing nursing students positive clinical learning environments with positive clinical instructors throughout their entire program may improve their intention to work in gerontological care settings.

There were a number of factors that influenced the study participants’ attitudes toward older adults and intention to work in a gerontological care setting, including their previous experience, clinical placements and their current workplace setting. Having previous work or volunteer experience in a gerontological care setting had a negative influencing effect on participant's intention to work in this specialty. This finding is similar to studies conducted with nursing students (Dahlke et al., 2019, 2020; Madueke et al., 2020; Stevens, 2011). Some participants in this study reported they worked as a PSW throughout nursing school and they felt that there were no additional learning opportunities in these facilities aside from providing basic care. This notion has been supported in the literature, whereby it has been suggested that only providing students with geriatric-focused clinical placement in their first year exposes them to the role of a PSW, and not the role of a nurse (Dahlke et al., 2020; Prentice, 2012).

The current workplace setting of nurse participants in this study also influenced their intention of a future career in gerontological nursing. Thirty-five percent of participants reported that they are currently working as a nurse in a setting where the majority of patients are over the age of 65 years. These participants were 20 times more likely to have a positive intention to work in a gerontological care setting within the next 5 years. However, 19% of participants who currently work in a gerontological care setting reported that they do not intend to continue working in this specialty in the next 5 years. This highlights the importance of determining how to successfully recruit and retain new nurses in gerontological care settings. Providing new nurses with mentoring programs focused on gerontological nursing after graduation may be one strategy to implement. The efficacy of a 12-month mentorship program has been examined in Australia and in the United States, and there was a substantial improvement in nurses’ retention rates, and in their feelings of self-efficacy to care for older adults in gerontological care settings (Lau et al., 2015; Salmond et al., 2017).

Only 14% of participants in this study had a positive intention to work in a gerontological care setting within the next 5 years, with RPNs being more likely to do so than RNs. Although there were no significant differences in gerontological content delivery methods, or attitudes toward older adults between RNs and RPNs, it is possible that the practical nursing curriculum may “socialize” RPNs toward a career in this field and setting more than the RN curriculum, with the prospect of increased job opportunities for RPNs in gerontological care settings (CNO, 2017; Haron et al., 2013; Lankshear & Rush, 2018). In any case, *all* nursing programs must encourage students to consider a future career in gerontological nursing. This approach is particularly important if educators wish to align with the recent recommendation from the Ontario “Public Inquiry into Safety and Security of Residents in the Long-Term Care Homes System” to increase the number of registered staff during the day, evening, and night shifts as a means to increase the safety of all residents (Gillese, 2019). This need for an increase in registered nursing professionals (RNs and practical nurses) in LTCFs highlighted in light of the global COVID-19 pandemic, and the illumination of the substantive system deficits in gerontological care settings. Now, more than ever, nurses who specialize in gerontological nursing are vital to the healthcare system and to provide care to a vulnerable population.

### Limitations

The nonexperimental, cross-sectional study design is a limitation of this study as cause and effect cannot be determined, and generalizability is limited. Other limitations include response rate and potential sampling bias. The response rate for this study was less than the 50% and not every question had the same response rate leading to a response bias. Sampling bias may have occurred given the homogeneity of the sample, and as a result of the snowball sampling method. In addition, there were 52 participants that were not asked the question “Most old people need no more love and reassurance than anyone else” from the KAOP scale which measures attitudes toward older adults. This was due to a printing error on the online survey. For participants who did not answer this question, the question was not included as part of their total score. This missing question may have altered the findings regarding attitudes regarding older adults. However, participants are asked this question as a negative as “Most old people make excessive demands for love and reassurance compared to anyone else” and this should help mitigate any negative impact of this missing question.

### Implications for nursing education, practice, and future research

The results of this study indicate that new nurses’ intentions to work in a gerontological care setting is most influenced by providing students with clinical placements in these settings over the course of their program. This is an important finding for educators and administrators in schools of nursing and gerontological care facilities. Nurse educators can provide students with a high quality gerontological nursing curriculum and comprehensive clinical placements to improve students’ intentions to work in these specialty care settings (McCleary et al., 2017). Including positive role models who have specialized in gerontology can improve students’ attitudes and intentions to work in gerontological care settings (Sheffler, 1998; Simpkins, 2013). Nurse managers in gerontological care settings should ensure that students feel welcome in these clinical practice environments. They can also offer final integrated practicum placements within their facilities to promote new nurses’ feelings of comfort in this environment and self-efficacy to work in this setting. Developing partnerships between nursing faculty and clinicians with a specialty in gerontological care can provide students with a variety of role models in this field and better prepare nurse graduates to care for the older adult population (Clendon, 2011).

Future research includes the need to understand how environmental and societal factors such as salary, workload, and societal ageism influences new nurses’ intentions to work in gerontological care settings. It is also important to understand how participants define a “gerontological nursing specialty” as many participants reported caring for adults over the age of 65 years but were currently working in acute care settings, such as medicine or cardiology units. Using qualitative research methodology may also be required to allow for an in-depth exploration of the nuanced factors that can improve nurses’ self-efficacy to care for the older adult population in gerontological care settings.

## Conclusion

This study provided a “snapshot” of how newly registered RNs and RPNs in Ontario, Canada perceive their gerontological nursing education, their attitudes toward older adults, and their future intentions of working in gerontological care settings. There was no significant relationship found between personal attitudes toward older adults and newly registered RNs’ and RPNs’ intentions to work in a gerontological care setting. This may indicate that having a positive, respectful attitude toward older adults is different than working as their care provider, and as such, must be conceptualized separately. A positive relationship was found between completing multiple geriatric-focused clinical placements as a student and newly registered RNs’ and RPNs’ intentions to work in a gerontological care setting. Therefore, enhancing the nursing curriculum to include more immersive gerontological-focused theory courses and multiple geriatric-focused clinical placements may increase the number of nurses intending to work in a gerontological care setting.

Few nurses in this study had definitive intentions to work in a gerontological care setting within the next 5 years. Nonetheless, there are other contributing factors which may impact new nurses’ intentions to work in gerontological care settings including salary, staffing ratios, and available support for new nurses in these facilities. Further research is needed to clarify the attitudes of new nurses’ in Ontario regarding working in these settings, and to determine how to encourage new nurses in Ontario toward a career caring for older adults. The shortage of nursing staff and the need for more nurses to work in gerontological care settings has been profoundly revealed during the COVID-19 pandemic. Research and changes to nursing curricula is needed to guarantee the care needs of one of our most vulnerable populations, older adults, are compassionately, safely, and effectively met and sustained.
